# Supplementary material for: Commiphora wildii Merxm. Essential Oil: Natural Heptane Source and Co-Product Valorization
Source: Molecules. 2023 Jan 16;28(2):891. doi: 10.3390/molecules28020891 (PMC9863203; doi:10.3390/molecules28020891)
Supplement: Supplementary file 1 [file molecules-28-00891-s001.zip › molecules-2134033-supplementary.pdf]

molecules

MDPI

Supplementary materials

Table S1. Chemical composition of *C. wildii* essential oil

Compounds	RI exp	RI theo	% GC
Unknown	677	-	0.2
**Heptane**	702	700	**29.5**
Methyl Isobutyl Ketone	735	738	tr
2-Methylheptane	757	765	tr
Toluene	763	760	0.1
Octane	800	800	0.3
2,4-Octadiene	805	815	tr
p-Xylene	867	866	tr
4-Heptanone	870	869	tr
**Compounds**	**Experimental RI**	**Theorical RI**	**%**
3-Heptanone	886	887	0.1
2-Heptanone	891	890-895	0.1
Nonane	900	900	0.9
4-Heptanol	897	-	0.1
3-Heptanol	903	892	0.1
2-Heptanol	907	900	0.1
Unknown	918	-	0.1
Tricyclene	920	925	0.1
α-Thujene	924	930-933	3.7
α-Pinene	934	932	43.4
Camphene	946	955	0.7
2,4-Thujadiene	950	949-951	0.4
Sabinene	970	969	0.5
β-Pinene	976	974	11.0
Unknown	988	-	0.1
2-Pentylfurane	989	990	0.1
α-Phellandrene	1002	1005-1008	0.1
α-Terpinene	1015	1008	0.4
p-Cymene	1018	1020	tr
Carvomenthene	1021	1021	tr
o-Cymene	1023	1023	2.3
Limonene	1027	1024	1.0
Eucalyptol	1030	1031-1036	0.1
m-Cymene	1033	1023-1026	0.1
(*Z*)-*β*-Ocimene	1035	1037-1038	0.1
(*E*)-*β*-Ocimene	1046	1048	0.2
γ-Terpinene	1056	1062	0.7
Sabinene hydrate*	1072	1070	0.1
α-Terpinolene	1084	1088	0.1
Unknown	1090	-	0.1
Unknown	1104	-	0.1
α-Thujone	1106	1105	tr
γ-Thujone	1118	1114	tr
Fenchol	1122	1112	tr
α-Campholene aldehyde	1127	1119	0.1

β-pinone	1139	1142	tr
Pinocarveol	1142	1134-1137	0.4
Camphre	1146	1147	0.2
Unknown	1149	-	0.2
Unknown	1153	-	0.1
Pinocamphone	1160	1154	0.1
Unknown	1162	-	0.1
Unknown	1175	-	0.2
Terpinen-4-ol	1181	1174	0.4
Myrtenal	1195	1193-1196	0.1
α-Terpineol	1198	1190	0.1
Myrtenol	1200	1194-1196	0.2
Verbenone	1209	1209	0.2
**Compounds**	**Experimental RI**	**Theorical RI**	**%**
Bornyl acetate	1283	1288	tr
cis-Verbenyl acetate	1290	1294	tr
Myrtenyl acetate	1323	1328	tr
β-Elemene	1387	1389	0.1
β-Caryophyllene	1415	1417	tr
Aromadendrene	1434	1436	tr
Guaiene	1485	1490	tr
α-Selinene	1492	1495	tr
δ-Cadinene	1516	1522	tr
Spathulenol	1576	1577	tr

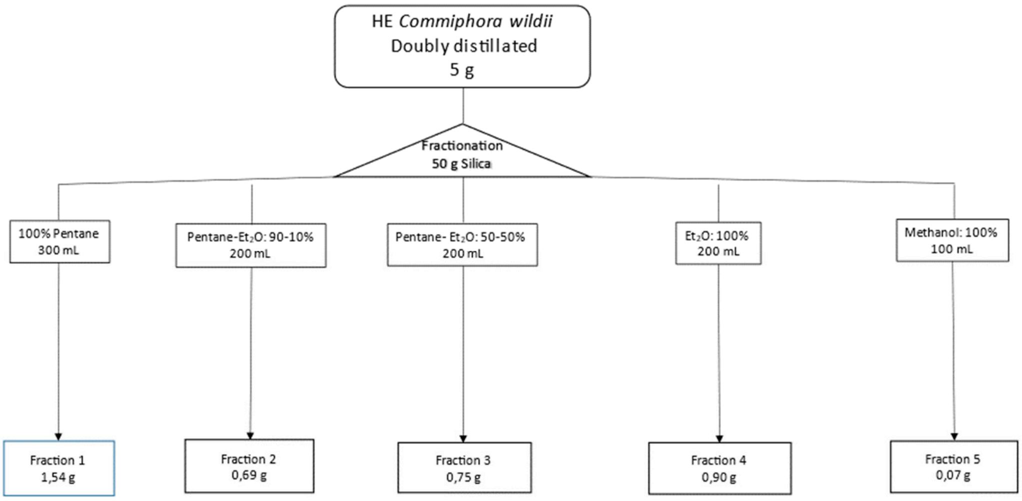

Figure S1. Fractionation protocol.

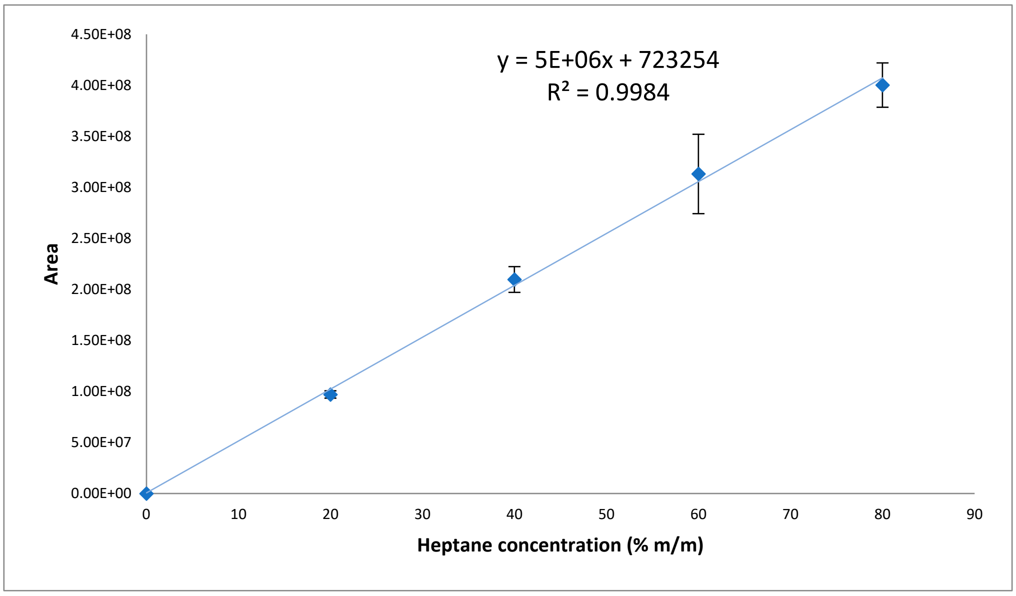

Figure S2. Heptane assay calibration curve.
